# Clinical algorithm reduces antibiotic use among children presenting with respiratory symptoms to hospital in central Vietnam

**DOI:** 10.1186/s41479-023-00113-9

**Published:** 2023-07-25

**Authors:** Phuong TK Nguyen, Tam TM Nguyen, Lan TB Huynh, Stephen M Graham, Ben J Marais

**Affiliations:** 1 Respiratory Department, Da Nang Hospital for Women and Children, Da Nang, Vietnam; 2 Sydney Vietnam Initiative, The University of Sydney, Sydney, Australia; 3 Centre for International Child Health, University of Melbourne and Murdoch Children’s Research Institute, Royal Children’s Hospital, Melbourne, Australia; 4 Discipline of Paediatrics and Adolescent Medicine, The Children’s Hospital at Westmead, Westmead, Australia; 5 Sydney Infectious Diseases Institute (Sydney ID), The University of Sydney, Sydney, Australia

**Keywords:** Child pneumonia, Vietnam, Hospital admission, Rational antibiotics, Implementation study

## Abstract

**Objective:**

To assess the safety and utility of a pragmatic clinical algorithm to guide rational antibiotic use in children presenting with respiratory infection.

**Methods:**

The effect of an algorithm to guide the management of young (< 5 years) children presenting with respiratory symptoms to the Da Nang Hospital for Women and Children, Vietnam, was evaluated in a before-after intervention analysis. The main outcome was reduction in antibiotic use, with monitoring of potential harm resulting from reduced antibiotic use. The intervention comprised a single training session of physicians in the use of an algorithm informed by local evidence; developed during a previous prospective observational study. The evaluation was performed one month after the training.

**Results:**

Of the 1290 children evaluated before the intervention, 102 (7.9%) were admitted to hospital and 556/1188 (46.8%) were sent home with antibiotics. Due to COVID-19, only 166 children were evaluated after the intervention of whom 14 (8.4%) were admitted to hospital and 54/152 (35.5%) were sent home with antibiotics. Antibiotic use was reduced (from 46.8% to 35.5%; *p* = 0.009) after clinician training, but adequate comparison was compromised. The reduction was most pronounced in children with wheeze or runny nose and no fever, or a normal chest radiograph, where antibiotic use declined from 46.7% to 28.8% (*p* < 0.0001). The frequency of repeat presentation to hospital was similar between the two study periods (141/1188; 11.9% before and 10/152; 6.6% after; *p* = 0.10). No child represented with serious disease after being sent home without antibiotics.

**Conclusions:**

We observed a reduction in antibiotic use in young children with a respiratory infection after physician training in the use of a simple evidence-based management algorithm. However, the study was severely impacted by COVID-19 restrictions, requiring further evaluation to confirm the observed effect.

## Background

Pneumonia is the leading cause of admission to paediatric wards in Vietnam [1, 2]. However, studies indicate that many admissions are not clinically indicated [1, 2] and that most children with likely viral infection receive unnecessary antibiotics [3]. Irrational use of intravenous broad-spectrum antibiotic in children with mild respiratory tract infection (ARI) symptoms and no clinical evidence of bacterial infection is a particular problem in Asian countries [3–5]. Excessive antibiotic use disturbs the normal microbiome and increases the risk of antimicrobial resistance, which may limit future treatment options. However, the unwanted effects of antibiotic use should be balanced against the risk of severe disease and death if bacterial pneumonia is not treated with appropriate antibiotics [3, 4, 6].

Physicians in central Vietnam generally adopt the practice of their superiors and formal guidelines on child respiratory tract infection management are infrequently updated and rarely followed. Many cases with uncomplicated viral infection are prescribed antibiotics [7], and physicians are usually reluctant to withhold antibiotics.

A major challenge to improve child pneumonia case-management is the development of a validated algorithm to guide clinical management in order to safely reduce irrational antibiotic use [8]. Such an algorithm should balance the need to limit excessive antibiotic use, and unnecessary hospitalisation, without putting the child at risk of adverse disease outcomes. In this study we assessed the effect of a single teaching session to encourage the use of a pragmatic management algorithm for reducing inappropriate antibiotic use in children with ARI presenting to a hospital outpatient department. This aligns with the fourth level of Kirkpatrick's model for assessing the effectiveness of training programs [9].

## Methods

We conducted a before-after intervention study at the Da Nang Hospital for Women and Children in Vietnam, a secondary referral hospital in the central region of Vietnam. The hospital also serves as a primary health care facility for people in its immediate surroundings. Children presenting with symptoms of ARI are routinely evaluated at the outpatient clinic. Since September 2020 a dedicated 24-h respiratory outpatient clinic, staffed by 5 doctors, was established to assess all children with respiratory symptoms and to conduct coronavirus disease 19 (COVID-19) screening. This respiratory outpatient clinic was selected for the intervention. In general, the clinic evaluated around 180 patients/day on weekdays and 100 patients/day on weekend days and we aimed to include at least 1200 patients in the pre- and 400 patients in the post-intervention period. However, this was reduced to less than 30 patients/day with the introduction of COVID travel restrictions in July 2021. At this time the clinic also evaluated more children referred from outlying district hospitals, many of whom were older than 5 years of age.

### Intervention

**Fig. 1:**
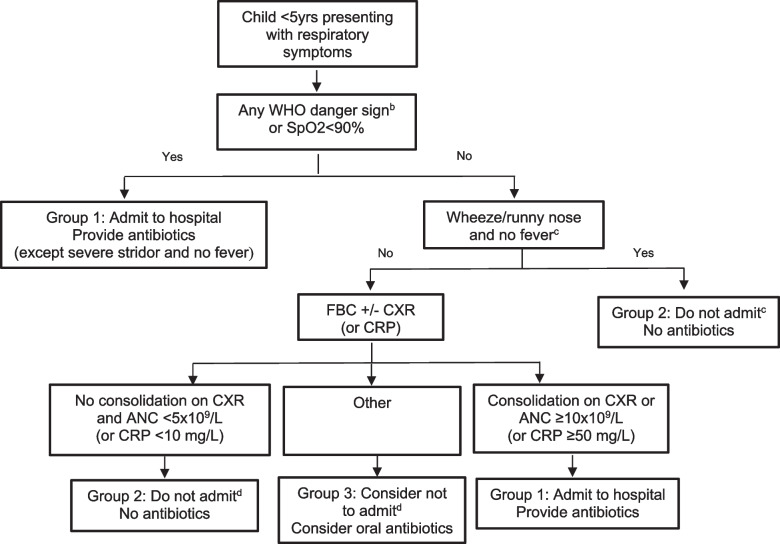
Pragmatic clinical algorithm for the management of children presenting to hospital with acute respiratory symptoms^a^ ANC – absolute neutrophil count; CXR – chest radiograph; FBC – full blood count; CRP: C reactive protein; SpO2—peripheral oxygen saturation; WHO – World Health Organization ^a^Incorporating study findings, existing WHO guidance and previous findings from Vietnam that used CRP values to guide rational antibiotic use [11]. ^b^Including inability to drink or breastfeed, vomiting everything, lethargic or reduced level of consciousness, convulsions, respiratory distress (grunting or nasal flaring), severe stridor, severe malnutrition. ^c^As per WHO recommendation [12]. ^d^Admit and consider antibiotics if any deterioration or relevant clinical concern

The intervention comprised a single training workshop where physicians were familiarised with the new clinical algorithm (Fig. 1). The development of this algorithm was informed by findings from a recent prospective observational study conducted at the same hospital over a one-year period [10]. Doctors were provided a detailed description of the rationale for the algorithm and how it was developed. As part of the intervention each examination room was provided with a copy of the algorithm (as a poster on the wall and a laminated A4 paper copy on the desk) and each doctor rehearsed the use of the algorithm under supervision of the study lead (PTKN). No other incentives or changes in practice were put in place during the study period. The post-intervention assessment was conducted one month after the training.

### Study population and data collection

We included children 2–59 months of age presenting to the outpatient clinic with respiratory symptoms. Since this was conducted as a clinical audit, data on all children were included unless they met exclusion criteria. We excluded children referred from other hospitals and those not presenting with respiratory symptoms or falling outside the specified age range. Children were triaged by nurses and then examined by doctors to determine if they (1) required immediate hospital admission, (2) required further laboratory or imaging tests or (3) could be discharged home, with or without prescription medication.

At baseline, we collected demographic data as well as clinical signs and symptoms. Clinical outcome, included admission to hospital, antibiotic prescription and type of antibiotic used. Any repeat presentation within one week of discharge was recorded. Study recruitment occurred over a three-month period on alternate days (Monday, Wednesday and Friday). The first month (22/02/2021 to 19/03/2021) provided the baseline, with clinicians following existing hospital practice. After training (as specified) we collected data over a two-month post intervention period (11/06/2021 to 13/08/2021) to record the impact of algorithm implementation. Algorithm use was not supervised and doctors could use or ignore it at their own discretion.

For the analysis, we classified children into one of two groups on the basis of either being admitted to hospital or discharged home, irrespective of whether additional investigations such as chest radiograph (CXR) and/or blood tests were performed. We documented the diagnosis (ICD-10 coding), management and outcome of all patients, as well as repeat hospital presentation within 1 week of discharge. Patients discharged home without hospital admission were contacted by telephone on day 4 and day 8 to check on their progress. Telephone follow-up was classified as unsuccessful if we were unable to contact parents after two attempts. In addition, patients in whom clinicians had any clinical concern were given a routine check-up appointment, which was counted as a representation in the ‘routine follow-up’ group.

### Statistical analysis

Data were entered into Epi data (version 4/4.2/1) and analysed using SPSS (version 24.0; SPSS, Inc., Chicago, IL). The chi square test was used to assess differences observed before and after the intervention; a Fisher’s exact test was used if numbers were less than 5. A p-value of less than 0.05 was considered statistically significant. We compared the percentage of children admitted to hospital before and after the intervention and assessed differences in their disease severity and spectrum, using ICD-10 coding. In addition, we compared the percentage of children who received antibiotics, including type and administration route (intravenous or oral). Potential risks associated with algorithm implementation were assessed by comparing the percentage of children who represented to hospital within 7 days of initial assessment.

## Results

**Fig. 2:**
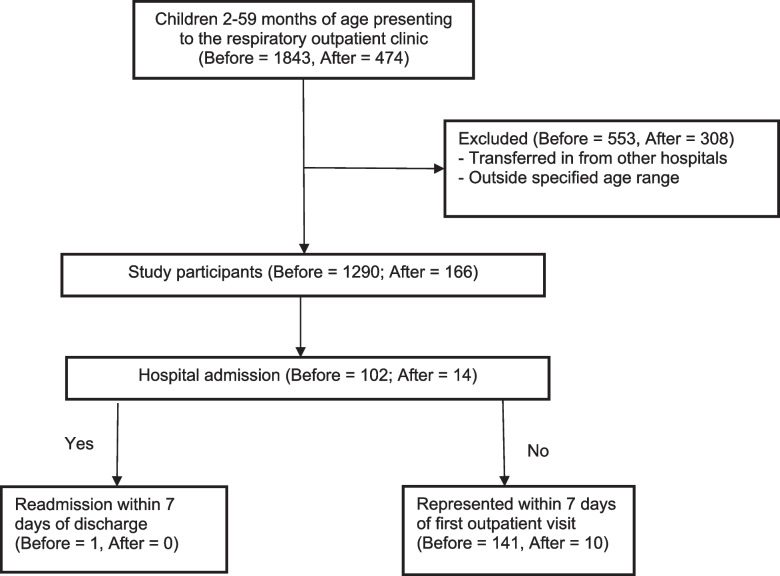
Diagram of study recruitment and hospital admission. CRF—case recording form; ICU – intensive care unit; ICD – international classification of diseases; CXR – chest X Ray; ANC – absolute neutrophil count. Before—data before implementation of the intervention. After—data after implementation of the intervention

Of 1,843 childen presenting to the clinic during the pre-intervention period, 1,290 (70,0%) were included. Unfortunately the number of children presenting to the clinic post-intervention was reduced to 474 due to COVID-19 restrictions, with only 166/474 (35.0%) included. Of 1,188 patients discharged during the pre-intervention period, 901 (75.8%) and 788 (66.3%) respectively were contacted via phone on day 4 and day 8 following discharge. These percentages were higher in the group discharged after the intervention; 150/152 (98.7%) on day 4 and 149/152 (98.0%) on day 8. Figure 2 illustrates study recruitment and hospital admission.

Table 1 summarises the demographic and clinical characteristics of children evaluated before and after the intervention. Relatively more children fell in the older (24–59 months) age category after the intervention (529; 41.0% before and 83; 50.0% after; *p* = 0.03). We observed a reduction in the number of CXR taken after the intervention (34.5% before and 14.5% after; *p* < 0.001). Interestingly, fewer patients were diagnosed with pneumonia (352, 27.3% before and 21, 12.7% after; *p* < 0.001) and more with viral upper ARI (658, 51.0% before and 108, 65.0% after; *p* = 0.001) after the intervention.

**Table 1:** Demographic and clinical characteristics of children who presented to the respiratory outpatient clinic, before and after the intervention

**Demographic and clinical characteristics at presentation**	**Before** *N* = 1290	**After** *N* = 166	***p*-value**
**Home address**			
Da Nang	611 (47.4)	106 (63.9)	< 0.001
Quang Nam^c^	493 (38.2)	42 (25.3)	0.001
Quang Ngai^c^	115 (8.9)	7 (4.2)	0.04
Other^c^	71 (5.5)	11 (6.6)	0.59
**Age**			
2–23 months	761 (59.9)	83 (50.0)	0.03
24–59 months	529 (41.0)	83 (50.0)	
**Signs and symptoms**			
Wheeze^a^	125 (9.7)	16 (9.6)	1.00
Runny nose	1010 (78.3)	140 (84.3)	0.09
Fever^b^	452 (35.0)	67 (40.4)	0.20
**Chest radiograph findings**			
Done	445/1290 (34.5)	24/166 (14.5)	< 0.001
Normal	262/445 (58.9)	20/24 (83.3)	0.02
Consolidation/patchy infiltration	106 (23.8)	3 (12.5)	0.32
Other	77 (17.3)	1 (4.2)	0.15
**Blood neutrophil**			
Done	518/1290 (40.2)	63/166 (38.0)	0.61
< 5,000 g/l	227/518 (43.8)	35/63 (55.6)	0.08
5–10,000 g/l	218 (42.1)	19 (30.2)	0.08
> 10,000 g/l	73 (14.1)	9 (14.3)	1.00
**CRP**			
Done	378/1290 (29.3)	55/166 (33.1)	0.32
< 50 g/l	335/378 (88.6)	47/55 (83.6)	0.27
≥ 50 mg/l	43 (11.4)	8 (14.4)	
**ICD-10 code assigned**			
Pneumonia (J15, J18)	352/1290 (27.3)	21/166 (12.7)	< 0.001
Bronchiolitis (J21)	85 (6.6)	4 (2.4)	0.04
Asthma (J45)	34 (2.6)	6 (7.6)	0.55
Bronchitis (J20)	161 (12.5)	27 (16.3)	0.18
URTI (J00-J06, H65, H66,J31)	658 (51.0)	108 (65.0)	0.001

*CRP* C reactive protein, *ICD* international classification disease, *URTIs* upper respiratory tract infections

^a^audible wheeze or wheeze on auscultation

^b^at presentation, temperature ≥ 38.5^0^C

^c^areas outside Da Nang that frequently use the outpatient services of the hosptial, formal hospital referrals were excluded

Table 2 reflects the clinical management and outcome of children not admitted to hospital. Overall the use of antibiotics was significantly reduced after algorithm training (46.8% before and 35.5% after; *p* = 0.009) with reductions mostly seen in younger children (aged 2–23 months). Before algorithm training 121/1290 (9.4%) children satisified discharged criteria of whom 76/121 (62.8%) received antibiotics, compared to 91/166 (54.8%) after algorithm training of whom 22/91 (24.4%) received antibiotics (*p* < 0.001). Of those without clinical risk signs who received antibiotics post-intervention, 7/22 (31.8%) had a C-reactive protein (CRP) ≥ 50 mg/dl (*n* = 3), absolute neutrophil count (ANC) ≥10.000/mm^3^ (*n* = 3), or abnormal CXR (*n* = 1).

**Table 2:** Clinical management and outcome of children with respiratory symptoms not admitted to hospital, before and after the intervention

**Management and outcome**	**Before**	**After**	***p*-value**
**Hospital admission**	102 (7.9)	14 (8.4)	0.76
**Discharged home**	***N* = 1188**	***N* = 152**	
**Phone call contact**			
Day 4	901 (75.8)	150 (98.7)	< 0.001
Day 8	788 (66.3)	149 (98.0)	< 0.001
**Antibiotic treatment**	556 (46.8)	54 (35.5)	0.009
Amoxicillin	74 (13.3)	4 (7.4)	0.29
Amoxicillin/clavulanate	247 (44.4)	25 (46.3)	0.89
Cefuroxime	92 (16.5)	15 (27.7)	0.06
Macrolide	110 (19.8)	9 (16.7)	0.72
Amoxicillin + macrolide	1 (0.2)	0	-
Amoxicillin/clavulanate + macrolide	25 (4.6)	0	-
Cefuroxime + macrolide	6 (1.0)	0	-
Clindamycin	1 (0.2)	0	-
Cephalexin	0	1 (1.9)	-
**Antibiotic treatment by age group**			
12–23 months	322 (57.9)	29 (53.6)	0.57
24–59 months	234 (42.1)	25 (46.4)	
**Representation within 1 week**	***N* = 141** (11.9)	***N* = 10** (6.6)	0.10
Reason revisit			
Prolonged cough	42 (29.8)	0	-
High fever^a^	33 (23.4)	7 (70.0)	0.004
Cough and fever	14 (9.9)	1 (10.0)	-
Breathlessness	4 (2.9)	1 (10.0)	-
Prolonged runny nose	2 (1.3)	0	-
Routine follow up by physician	19 (13.5)	1 (10.0)	-
Other	27 (19.2)	0	-
***Diagnosis at representation***			
Pneumonia	45 (31.9)	1 (10.0)	
Asthma	7 (5.0)	2 (20.0)	-
Bronchiolitis	6 (4.3)	0	-
Bronchitis	8 (5.7)	0	-
URTI	58 (41.1)	2 (20.0)	-
Others (viral infections)	17 (12.0)	5 (50.0)	-
***Antibiotic treatment at representation***	70 (49.6)	2 (20.0)	
Macrolide	8 (11.4)	1 (50)	-
Cefuroxime	13 (18.6)	1 (50)	-
Amoxicillin	1 (1.4)	0	-
Amoxicillin/clavulanate	38 (5.3)	0	-
Amoxicillin/clavulanate + macrolide	6 (8.6)	0	-
Cefuroxime + macrolide	4 (5.7)	0	-
***Hospital admission at representation***	***N* = 25 (17.7)**	***N* = 3 (30.0)**	0.40
Admitted to DHWC	18 (72.0)	2 (66.7)	1.00
Admitted to another hospital	7 (28.0)	1 (33.3)	-

*URTI* upper respiratory tract infection, *DHWC* the Da Nang Hospital for Women and Children

^a^subjective fever as reported by the parent

The percentage of children with repeat hospital presentation within one week was not increased after the intervention (11.9% before and 6.6% after; *p* = 0.10). Of the 10 children who represented to hospital following outpatient discharge in the post intervention period, one had a routine follow-up, seven had ongoing fever (one with additional diarrhoea), one had new signs of hand foot and mouth disease and one had persistent wheezing. The child with persistent wheezing is the only case that developed World Health Organisation (WHO) danger signs, classified as severe respiratory distress in the absence of fever. Only two of the children with persistent fever received antibiotics, none had danger signs and most (5/7; 71.4%) resolved without treatment. On telephone review none of the children reported clinical deterioration on day 4 or day 8 after hospital discharge.

Table 3 compares the clinical management and outcome of hospitalised children. Hospital admission rates were similar during the two periods (7.9% before and 8.4% after; *p* = 0.76), but there was a significant reduction in antibiotic use during hospital admission; from 32/36 (88.9%) to 8/13 (61.5%; *p* = 0.04). Table 4 compares the management of children presenting with respiratory symptoms, according to their algorithmm classification, as described in Fig. 1. The greatest reduction in the use of antibiotics was observed in group 2, consisting of children with a wheeze or runny nose and no fever, or those with a CXR or blood test results not indicative of bacterial infection (ANC < 5 × 10^9^/L or CRP < 10 mg/L). In these children the algorithm suggests discharge home without antibiotics and the use of antibiotics decreased significantly, from 46.7% before to 28.8% after the intervention (*p *< 0.0001).

**Table 3:** Clinical management and outcome of children with respiratory symptoms who were hospitalised, before and after the intervention

**Management and outcome**	**Before** *N* = 38	**After** *N* = 13	***p*-value**
**Department admitted to**			
Infectious diseases	36 (94.7)	12 (92.3)	-
Respiratory	2 (5.3)	1 (7.7)	-
Reason for admission*			
Underlying co-morbid conditions^a^	5 (12.2)	0	-
Parental worries	1 (2.4)	0	-
Unresponsive to first line treatment	19 (46.3)	8 (61.5)	-
Danger signs^b^ and Sp02 < 92%	7 (17.0)	1 (7.7)	-
Others^c^	9 (22.1)	4 (30.8)	-
**Clinical signs and symptoms**			
Fast breathing^d^	24 (63.2)	5 (38.5)	0.19
Sp02 < 92%	7 (18.4)	1 (7.7)	0.66
Danger signs^b^	2 (5.3)	1 (7.7)	1.00
Fever^e^	24 (63.2)	10 (76.9)	0.50
Wheeze^f^	10 (26.3)	2 (15.4)	0.71
**Chest radiograph findings**	38 (100.0)	13 (100.0)	-
Normal	13 (34.2)	7 (53.8)	0.32
Consolidation/patchy infiltration	16 (42.1)	2 (15.4)	0.10
Other abnormalities	9 (23.7)	4 (30.8)	0.72
**Blood neutrophil count**	38 (100.0)	13 (100.0)	-
< 5,000 g/l	13 (34.2)	7 (53.8)	0.32
5–10,000 g/l	15 (39.5)	3 (23.1)	0.34
> 10,000 g/l	10 (26.3)	3 (23.1)	1.00
**CRP**	36 (94.7)	13 (100.0)	-
< 50 g/l	30 (83.3)	10 (76.9)	0.68
≥ 50 mg/l	6 (16.7)	3 (23.1)	
**Diagnosis at admission**			
Pneumonia	25 (65.8)	6 (46.2)	0.32
Asthma	1 (2.6)	0 (0)	-
Bronchiolitis	9 (23.7)	1 (7.6)	-
URTI	3 (7.9)	6 (46.2)	< 0.001
**Diagnosis at discharge**			
Pneumonia	24 (63.2)	6 (46.2)	0.34
Asthma	1 (2.6)	0 (0)	-
Bronchiolitis	8 (21.1)	0 (0)	-
URTI	5 (13.1)	7 (53.8)	< 0.001
**Treatment**			
Oral antibiotics	26 (72.2)	7 (53.8)	0.50
IV antibiotics	6 (16.7)	1 (7.6)	0.66
No antibiotics	4 (11.1)	5 (38.6)	0.04
**Readmission within 1 week**	1 (2.6)	0 (0)	-

*Sp02* peripheral oxygen saturation, *CRP* C-reactive protein. *URTIs* upper respiratory tract infections, *IV* intravenous

^*^More than 1 reason possible

^a^such as congenital heart disease, or immunocompromise

^b^inability to breastfeed or drink from bottle, vomiting everything, lethargy or reduced level of consciousness, convulsions, respiratory distress (grunting or nasal flaring), severe stridor, severe malnutrition

^c^high fever and cough, abnormal blood test results defined as breath rate of ≥ 50/minute aged 2–11 months, or ≥ 40/minute aged 12–59 months [13]

^e^temperature ≥ 38.5^0^C on admission

^f^audible wheeze or wheeze on auscultation

**Table 4:** Management of children presenting to the respiratory outpatient clinic with respiratory symptoms according to algorithm classification, before and after the intervention

Classification*	**Before** *N* = 1290	**After** *N* = 166	***p*-value**
**Group 1**^**a**^			
Number	22	6	-
Admitted to hospital	6 (27.3)	1 (16.7)	-
Discharged with antibiotic	13 (59.1)	4 (66.6)	0.1
Discharged without antibiotic	3 (13.6)	1 (16.7)	
**Group 2**^**b**^			
Number	355	118	-
Admitted to hospital	14 (3.9)	2 (1.7)	-
Discharged with antibiotic	166 (46.7)	34 (28.8)	< 0.0001
Discharged without antibiotic	175 (49.4)	82 (69.5)	
**Group 3**^**c**^			
Number	913	42	-
Admitted to hospital	82 (8.9)	11 (26.2)	-
Discharged with antibiotic	383 (41.9)	18 (42.9)	0.2
Discharged without antibiotic	448 (49.2)	13 (30.9)	

^*^Classified according to groups described in Fig. 1 from retrospective data analysis

^a^Children with any WHO danger sign or SpO2 < 90% OR consolidation on CXR or ANC ≥ 10 × 10^9^/L (or CRP ≥ 50 mg/L). Suggested management: admit to the hospital and provide antibiotics

^b^Children with wheeze/runny nose and no fever OR No consolidation on CXR and ANC < 5 × 10^9^/L (or CRP < 10 mg/L). Suggested management: no hospital admission and no antibiotics

^c^Children not belonging to groups 1 or 2. Suggested management: consider not to admit and consider oral antibiotics

*ANC* absolute neutrophil count, *CXR* chest radiograph, *FBC* full blood count, *CRP* C reactive protein, *SpO2* peripheral oxygen saturation, *WHO* World Health Organization

## Discussion

We observed a significant reduction in antibiotic use in young children with a respiratory infection after physician training in the use of a simple evidence-based management algorithm. However, the study was greatly impacted by COVID-19 restrictions, which affected the numbers recruited to the post-intervention period. Importantly, the reduced use of antibiotics was not associated with increased risk for disease progression or representation to hospital during active follow-up. Given that many children with respiratory symptoms in Vietnam and Asia receive antibiotics without a strong clinical indication [1, 14, 15], implementation of this algorithm may provide clinicians with a practical method to re-evaluate established practices and to encourage more judicious antibiotic use.

Studies in both African [16] and Asian settings [17] have demonstrated that wheeze is strongly associated with asthma or viral infections. Similar to the algorithm used in the intervention, revised WHO guidance for community acquired pneumonia recommends that a child with wheeze and no fever or danger signs, should not receive antibiotic treatment [12]. Digital auscultation used in the multi-centre PERCH study, conducted in seven Asian and African countries, reported low mortality and reduced likelihood of radiographic pneumonia in children with an audible wheeze [18]. In settings where special tests are available, a CXR and full blood count and/or CRP could provide clinicians with additional confidence to withhold antibiotics in a child with respiratory symptoms [11, 19].

Physicians in Asian countries prefer to have radiology and blood test results to guide the clinical management of children with ARIs [5, 11]. Previous studies have identified a raised neutrophil count (≥ 10 × 10^9^/L) and an abnormal CXR as markers of potential bacterial pneumonia [20–22], but the specificity is low. Although dense alveolar consolidation on CXR shows a consistent association with bacterial pneumonia [23, 24], these findings are often influenced by inclusion bias if CXR interpretation influenced disease classification and some studies have questioned the strength of the association [25]. In the PERCH study, dense alveolar consolidation on CXR or the presence of pleural fluid were associated with *Streptococcus* pneumoniae or *Staphylococcus* aureus infection, but it was also observed in children who only had proof of a viral infection [22]. The management algorithm used a CRP cut-off of 50 mg/l, since a randomised controlled trial in Vietnam demonstrated that it is safe to withhold antibiotics in children with acute lower respiratory tract infections if the CRP is < 50 mg/l [11].

It was hoped that the management algorithm would also reduce unnecessary hospital admission, but this could not be demonstrated in the current study. Due to parental pressure and hospital policy preference, clinicians often feel that hospitalisation is the ‘safe option’ [5]. However, unnecessary hospitalisation poses many risks and increases health service costs [26, 27]. Recent studies have shown that Vietnam is rapidly transitioning to become a middle income country with low child mortality [28] with bacterial pneumonia rates more comparable to high-income settings [29–32]. A common perception among health workers is that the WHO clinical case-management approach for childhood pneumonia was developed for low-income countries, which is not applicable to Vietnam [33]. Hence, an algorithm that differentiates children who present to hospital with ‘unlikely bacterial pneumonia’ from those with ‘likely bacterial pneumonia’, and which takes CXR and blood test results into account, has more appeal in settings where these tests are readily available [33].

The ‘WHO danger signs’ was the strongest predictor of pneumonia mortality in the PERCH study [34] and ‘consolidation on CXR have also been shown to be a strong predictor of ‘adverse pneumonia outcome’ in Vietnam [10]. We incorporated both these factors in the algorithm and have shown that their consideration is highly feasible in a hospital-based setting, where CXR findings provide clinicians with another important line of information and reduces parental anxiety [35]. Given the sharp decrease in the number of hospital presentations and the change in patient profile due to the COVID-19 lockdown, we could not assess the impact of algorithm training on hospital admisison rates. Perceived parental pressure and physician’s reluctance to miss potentially serious disease have been reported as the main drivers of unnecessary antibioitc use in Vietnam [7].

There are major study limitations to emphasise. Firstly, patient numbers after the intervention were greatly reduced, due to strict COVID-19 lockdowns implemented during this time. We acknowledge that the epidemiology of other ARIs may also have changed, due to strict COVID-19 social distancing and health system disruption [36, 37], as well as effects of the SARS-CoV-2 virus [38]. Although clinical symptoms were broadly comparable between the two periods, more children were excluded after the intervention, reflecting the fact that more older children were refered from district hospitals during this time. However, the children included after the intervention were expected to be a sicker cohort, which would have biased against observing a signficant reduction in antibiotic use. Secondly, less CXRs were done during COVID-19 restrictions, but again the expectation is that this would have increased antibiotic use if physicians felt that it was more difficult to rule out bacterial pneumonia. Data collection for the baseline study were collected during the winter-spring season, whereas the two-month intervention period occurred during the summer season, however, Vietnam does not display clear seasonal patterns or pronounced seasonal variability in the frequency of acute respiratory tract infections [1].

In conclusion, we observed a significant reduction in antibiotic use among children with ARIs after doctors were trained in the use of a simple management algorithm and no major risks were documented. It is prudent to note that given the confounding impact of the COVID-19 pandemic that emerged during the study period, our findings need to be interpreted with caution and further evaluation to confirm the impact and safety of the proposed intervention is required.

## Data Availability

The datasets used and/or analysed during the current study are available from the corresponding author on reasonable request.

## Abbreviations

ANC: Absolute neutrophil count
ARI: Respiratory tract infection
COVID: Coronavirus disease
CRP: C-reactive protein
CXR: Chest radiograph
ICD: International classification of diseases
SARS-CoV-2: Severe acute respiratory syndrome coronavirus 2
SPSS: Statistical package for the social sciences
WHO: World Health Organisation
